# Solitary mediastinal lymph node metastasis in rectosigmoid carcinoma: a case report

**DOI:** 10.1186/1757-1626-1-69

**Published:** 2008-07-31

**Authors:** Khaled M Musallam, Ali T Taher, Ayman N Tawil, Zaher I Chakhachiro, Moh'd Z Habbal, Ali I Shamseddine

**Affiliations:** 1 Department of Internal Medicine, Hematology-Oncology Division, American University of Beirut Medical Center, Beirut, Lebanon; 2 Department of Pathology and Laboratory Medicine, American University of Beirut Medical Center, Beirut, Lebanon

## Abstract

**Introduction:**

Colorectal cancer most commonly metastasizes to the regional lymph nodes, liver, bone, lung, and brain. Metastases to mediastinal lymph nodes is a rare entity which has never been reported to be solitary.

**Case report:**

We herein describe a 67-year-old male patient with a solitary mediastinal lymph node metastasis three years following the resection of his primary rectosigmoid carcinoma. Pathological characteristics of the metastatic tissue and technical limitations in imaging modalities resulted in incongruity between follow-up CT and PET scans. Diagnosis of this distant metastasis has been confirmed through a mediastinoscopic biopsy.

**Conclusion:**

Attention should be paid to the mediastinum when evaluating PET scan or CT films during follow-up of patients with colorectal cancer. Using PET/CT instead of separate morphological and functional data sets favors better detection. Questions still remain concerning the ideal management protocol of such a presentation, the two main options being locoregional or chemotherapeutic.

## Introduction

Colorectal cancer is the third most common cancer as well as the third most common cause of cancer death in both men and women [1]. Rectosigmoid colon continues to be the most common site while the cecum, ascending and transverse colon are increasingly reported. Colorectal cancer most commonly metastasizes to the regional lymph nodes, liver, bone, lung, and brain. Rare metastases to mediastinal lymph nodes from colonic carcinomas have also been reported [2-5]. Postulated mechanisms of spread were lymphatic drainage routes of the liver in those with concurrent liver metastases; and paravertebral venous or paraaortic lymphatic plexus in those with concomitant pelvic or abdominal metastases. Mediastinal lymphadenopathy, attributed to coexisting sarcoidosis, has also been reported in one case of colon cancer [6]. To our knowledge, our report is the first in the literature to demonstrate solitary mediastinal lymph node metastases of colorectal carcinoma with no other site involvement.

## Case Report

**Figure 1:**
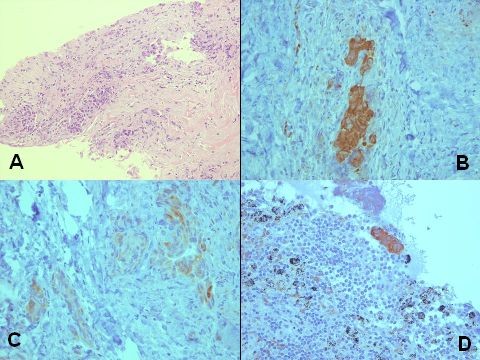
**Colonic tissue in paratracheal nodes**. Paratracheal nodal involvement by metastatic adenocarcinoma (A) [H&E, 200×]. The tumor cells are strongly positive for CK20 (B) [400×], weakly positive for CK7 (C) [400×]. Micrometastasis in another paratracheal lymph node (D) [400×].

**Figure 2:**
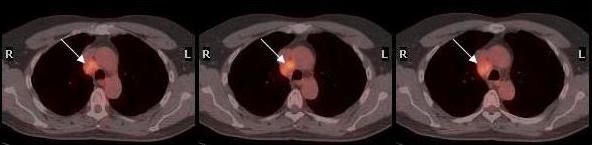
**PET scan of thorax**. ^18^F-FDG PET scan of the thorax showing a 2.2 cm enlarged right paratracheal lymph node.

We describe the case of a 67-year-old man with evidence of right paratracheal lymph node metastasis three years following the diagnosis of rectosigmoid carcinoma. In October 2004, the patient initially presented with symptoms and signs of intestinal obstruction. Computed tomography (CT) scan of the abdomen and pelvis showed diffuse circumferential intramural thickening with associated streaking of the adjacent fat planes in a short segment of the sigmoid colon. Sigmoidoscopic biopsy revealed an infiltrating moderately differentiated adenocarcinoma. Subtotal colectomy was eventually performed. Pathologic examination of the resected tissue revealed a five centimeter tumor invading the muscularis propria and pericolonic fat. Metastatic adenocarcinoma was noted in ten out of fifty peri-tumoral and mesenteric lymph nodes with extracapsular extension. The patient was eventually diagnosed with stage III adenocarcinoma of the rectosigmoid colon and given six cycles of adjuvant chemotherapy. In December 2006, a follow up total body CT scan identified an enlarged 1 × 1 cm right paratracheal lymph node, with no evidence of pulmonary infiltrates, masses or fluid in the pleural cavities. There were no other significant findings in the pelvis or abdomen. The recommendation was to perform a fluor-18-deoxyglucose-positron emission tomography (^18^F-FDG PET) scan, which did not show any evidence of active disease; and hence, tumoral metastasis was ruled out and no action was taken. In October 2007, the previously identified lymph node appeared to have increased in size to approximately 2.2 cm, with the remainder of the CT scan being insignificant. Mediastinoscopic biopsy of the lymph node was done and revealed metastatic adenocarcinoma, similar to that of the colon (Figure 1A; ×200). Biopsy of another paratracheal lymph node showed two cell clusters of metastatic adenocarcinoma that were only identified by immunohistochemical analysis (Figure 1B; ×400). The metastatic tumor was positive for cytokeratin AE1/AE3 and cytokeratin 20 (Figure 1C; ×400), weakly positive for cytokeratin 7 (Figure 1D; ×400), and negative for thyroid transcription factor-1 (TTF1), confirming its colonic origin. Consequently, a total body ^18^F-FDG PET scan was requested which now identified the lymph node (Figure 2), and revealed no other significant findings. All through out the follow-up period the patient never had any respiratory or gastrointestinal symptoms. Ca19-9 and CEA serum levels were always within the normal range.

## Discussion

We propose the following explanations for the incongruity between the follow-up imaging studies performed on the patient described in this report. ^18^F-FDG PET scan has proven superior to morphologic imaging procedures when assessing lymph node involvement based on functional data evaluating tumor metabolism [7]. A meta-analysis of the most recent literature showed that changing the therapy plan by the use of ^18^F-FDG PET scan in patients with colorectal metastatic cancer occurs in 31.6% of the cases [8]. However, small malignant lesions may not show increased tracer uptake since current PET-detectors provide an in-plane spatial resolution of only 4–5 mm. This limitation is most heightened in the thorax where lesion detection on PET is further compromised by respiratory motion (shallow breathing during PET acquisition) [9]. Furthermore, the extensive fibrosis evident by pathological examination of the mediastinal lymph node in our case might have led to a decrease in uptake of ^18^F-FDG during the initial PET scan in April 2006. Several studies, evaluating patients with different oncological diseases, have reported outstanding results concerning tumor staging when using PET/CT instead of separate morphological and functional data sets [10,11].

Upgrading from stage III to stage IV ensues after detection of this distant metastasis. However, one question still remains: should the management of this patient rely on systemic chemotherapy only or could the patient benefit from loco-regional radiation and/or surgical resection? The rationale would be that resection or radiation of the mediastinal lymph node may prevent possibly fatal complications caused by tracheal and major vascular involvement; however, no evidence to support this reasoning currently exists.

## Conclusion

Our case report suggests that attention should be paid to the mediastinum when evaluating PET scan or CT films during follow-up of patients with colorectal cancer. Moreover, despite the fact that the ^18^F-FDG PET scan has high specificity for colorectal metastases; all suspected lesions, identified though morphological imaging, must be further investigated if they can change the therapeutic plan. However, the choice between locoregional and chemotherapeutic subsequent management remains unclear in the case of solitary mediastinal metastasis.

## Consent

Written informed consent was obtained from the patient for publication of this Case report and any accompanying images. A copy of the written consent is available for review by the Editor-in-Chief of this journal.

## Abbreviations

^18^F-FDG PET: 18-F-fluorodeoxyglucose positron emission tomography; CT: Computed tomography; TTF1: Thyroid transcription factor-1.

## Competing interests

The authors declare that they have no competing interests.

## Authors' contributions

KMM designed the study, compiled the different sections of the report, and helped in writing the manuscript. AIS and ATT drafted the manuscript and revised it critically for important intellectual content. ANT, ZIC, and MZH helped in preparing the images and writing the pathology sections. All authors read and approved the final manuscript.
